# Neurofilament degradation is involved in laparotomy-induced cognitive dysfunction in aged rats

**DOI:** 10.18632/aging.104172

**Published:** 2020-11-24

**Authors:** Yiyun Cao, Taotao Liu, Zhengqian Li, Jiao Yang, Lijun Ma, Xinning Mi, Ning Yang, Aihua Qi, Xiangyang Guo, Aizhong Wang

**Affiliations:** 1 Department of Anesthesiology, Sixth People’s Hospital Affiliated to Shanghai Jiao Tong University, Shanghai 200233, China; 2 Department of Anesthesiology, Peking University Third Hospital, Beijing 100191, China; 3 Department of Pharmacy, Sixth People’s Hospital East Campus Affiliated to Shanghai Jiao Tong University, Shanghai 200233, China; 4 Department of Medical Imaging, North Minzu University, Yinchuan, Ningxia 750021, China

**Keywords:** postoperative cognitive dysfunction (POCD), neuroinflammatory, laparotomy, neurofilaments, trichostatin A

## Abstract

Excessive neuroinflammatory responses play important roles in the development of postoperative cognitive dysfunction (POCD). Neurofilaments (NFs) were essential to the structure of axon and nerve conduction; and the abnormal degradation of NFs were always accompanied with degenerative diseases, which were also characterized by excessive neuroinflammatory responses in brain. However, it is still unclear whether the NFs were involved in the POCD. In this study, the LC-MS/MS method was used to explore the neuroinflammatory response and NFs of POCD in aged rats. Moreover, trichostatin A (TSA), an inflammation-related drug, was selected to test whether it could improve the surgery-induced cognitive dysfunction, inflammatory responses and NFs. Evident cognitive dysfunction, excessive microglia activation, neuroinflammatory responses and upregulated NFs in hippocampus were observed in the POCD group. TSA pretreatment could significantly mitigate these changes. The KEGG analysis revealed that nine pathways were enriched in the TSA + surgery group (versus the surgery group). Among them, two signaling pathways were closely related with the changes of NFs proteins. In conclusion, surgery could impair the cognitive function and aggravate neuroinflammation and NFs. The TSA could significantly improve these changes which might be related to the activation of the “focal adhesion” and “ECM-receptor interaction” pathways.

## INTRODUCTION

Postoperative cognitive dysfunction (POCD) commonly occurs in aged patients after surgery and is easily under-recognized, which is always accompanied with longer hospital stays, decreased quality of life, increased mortality and risk of dementia. In recent years, many studies have suggested that the pathogenesis of POCD is a synergistic effect of various factors, including tau phosphorylation, amyloid β accumulation [1], blood brain barrier (BBB) disruption [2] and immune system disorders [3, 4]. However, the precise pathophysiology of POCD remains unclear.

Microglia constitute 10%–15% of the glial cell population in the parenchyma in the adult brain [5]. Dysfunction of microglia is involved in the amplification of the immune response in the pathophysiology of neurological disorders, including ischemic stroke [6] and neurodegenerative diseases [7, 8]. Overexpression of inflammatory cytokines in the brain after surgery and anesthesia [3, 4] can be attributed to excessive activation of microglial [9]. Our previous studies revealed that laparotomy under Isoflurane, an established POCD model [10, 11], could increase the proinflammatory cytokines interleukin-1β (IL-1β) and tumor necrosis factor-alpha (TNF-α) in the hippocampus and cause cognitive dysfunction in aged rats. Interactions between neuroinflammatory and neuronal components are important for normal CNS function, but its extensive cross-reaction in POCD hippocampal neurons remain elusive and need to be further explored.

Adult neurons in the central nervous system (CNS) express the pan-neuronal type IV intermediate filaments (NF triplet proteins: light, middle and heavy; henceforth called NEFL, NEFM, NEFH; and α-internexin) [12]. NFs are present in the neuronal cytoskeleton, especially the axon, are mainly responsible for increasing and maintaining axonal caliber and therefore improving relay of electrical impulses along the axons [13]. NF aggregation, however, has been convincingly demonstrated contribute to hinder axonal transport and impair the sub-cellular distribution of vesicles and mitochondria in rat primary neurons and neuronal cell lines overexpressing mutant NF proteins [14, 15]. Studies have shown serum and CSF NEFL levels are high in AD [16] and ischemic stroke [17] cases than in healthy controls. NEFL has also been used as a biomarker for cognition impairment in AD [18] and in Down syndrome to predict dementia status [19]. Previous study has explored that the microglial activation interactions with injured axonal swelling following traumatic brain injury in the micro pig [20].

Trichostatin A (TSA), a histone deacetylase (HDAC) inhibitor, was used to explore histone hyperacetylation in the transcriptional regulation of c-fos and c-jun genes in neuronal cells [21]. It can also act on nonhistone proteins which in turn may affect the migration of microglia and suppress the expression of both pro- and anti-inflammatory activation markers in microglia [22, 23], but its role in surgery-induced neuroinflammation in aged brains is unclear. Hence, our study explored whether TSA can reduce microglia-mediated neuroinflammation and improve surgery-induced cognitive dysfunction as well as the possible mechanism.

In the present study, we examined POCD following laparotomy in aged rats. Furthermore, we examined hippocampal microglial activation, proinflammatory cytokines release in producing the cognitive decline in aged rats following surgery by administering TSA or not. Peaking of neuroinflammation, the isobaric tags for the relative and absolute quantitation (iTRAQ) method combined with nano liquid chromatography-mass spectrometry (NanoLC-MS/MS), developed for protein quantitation, were used to reveal the profile of differentially altered proteins in the hippocampus underlying the animal model of POCD, and bioinformatics analyses were performed to explore surgery-induced regulated proteins and the correlated signaling pathway in the surgery + TSA *vs.* the surgery group. The study aimed to reveal the pathogenesis of POCD and provide potential therapeutic targets for its prevention and treatment in the vulnerable older brain.

## RESULTS

### Physiological parameters following the laparotomy protocol

There were no significant differences in the arterial blood gas values and blood glucose concentrations (Table 1) among the experimental groups immediately after laparotomy. These results reduce the possibility that the surgery protocol of the current study caused physiologic side effects of hypoxia, hypercapnia and hypoglycemia.

**Table 1:** TSA preconditioning and surgery administration have no effect on arterial blood gas values of aged rats.

**Group**	**pH**	**PO_2_(mmHg)**	**PCO_2_(mmHg)**	**Glucose(mmol/L)**
control	7.40 ± 0.15	80.2 ± 3.71	38.6 ± 1.50	5.9 ± 0.76
surgery	7.39 ± 0.18	81.1 ± 3.16	38.4 ± 1.38	6.3 ± 0.71
surgery + TSA	7.41 ± 0.21	80.8 ± 4.01	38.3 ± 1.49	6.0 ± 0.87

Data are shown as the mean ± standard error (n = 4 each group) (*P* > 0.05).

surgery: laparotomy; TSA, trichostatin A.

### TSA pretreatment improves cognitive function after surgery

**Figure 1:**
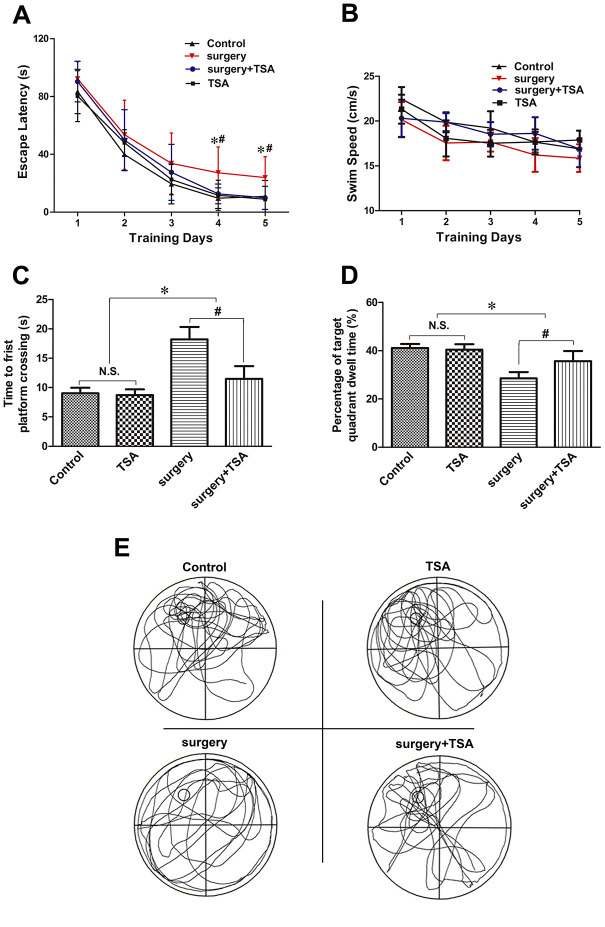
**TSA can mitigate surgery-induced spatial learning and memory impairments in aged rats.** (**A**, **B**) Acquisition trials demonstrating latencies for rats to locate the hidden platform during the 5 testing days (**A**) and the swimming speed (**B**), measuring spatial information acquisition. (**C**, **D**) On test day 6, probe trials demonstrating the time to first platform crossing (**C**) and time spent in the target quadrant (**D**), measuring memory retention capabilities. (**E**) Representative searching swimming paths of four aged rats with different treatments in the probe trial tests. Data are given as means {plus minus} SEM, n = 12. *P < 0.05 vs. the control group; #P < 0.05 vs. the surgery group.

During the MWM test, we found that aged rats in the surgery group showed longer escape latencies than those in the control group, the surgery + TSA group and TSA group on days 4 and 5 postsurgery (Figure 1A; *P* < 0.05); No significant difference was observed in latencies among the control, the surgery + TSA group and TSA groups. All aged rats appeared to swim normally, and swimming speeds were not different among groups (Figure 1B; *P* > 0.05). In the probe test, rats in the surgery group required a longer time to achieve their first platform crossing (Figure 1C; *P* < 0.05), and the time spent in the target quadrant was much shorter than that of rats in the control group (Figure 1D, 1E; *P* < 0.05), confirming the presence of memory impairments after surgery. All of these changes were significantly alleviated by TSA pretreatment, suggesting that TSA improves cognition.

### TSA inhibits surgery-induced microglia activation

**Figure 2:**
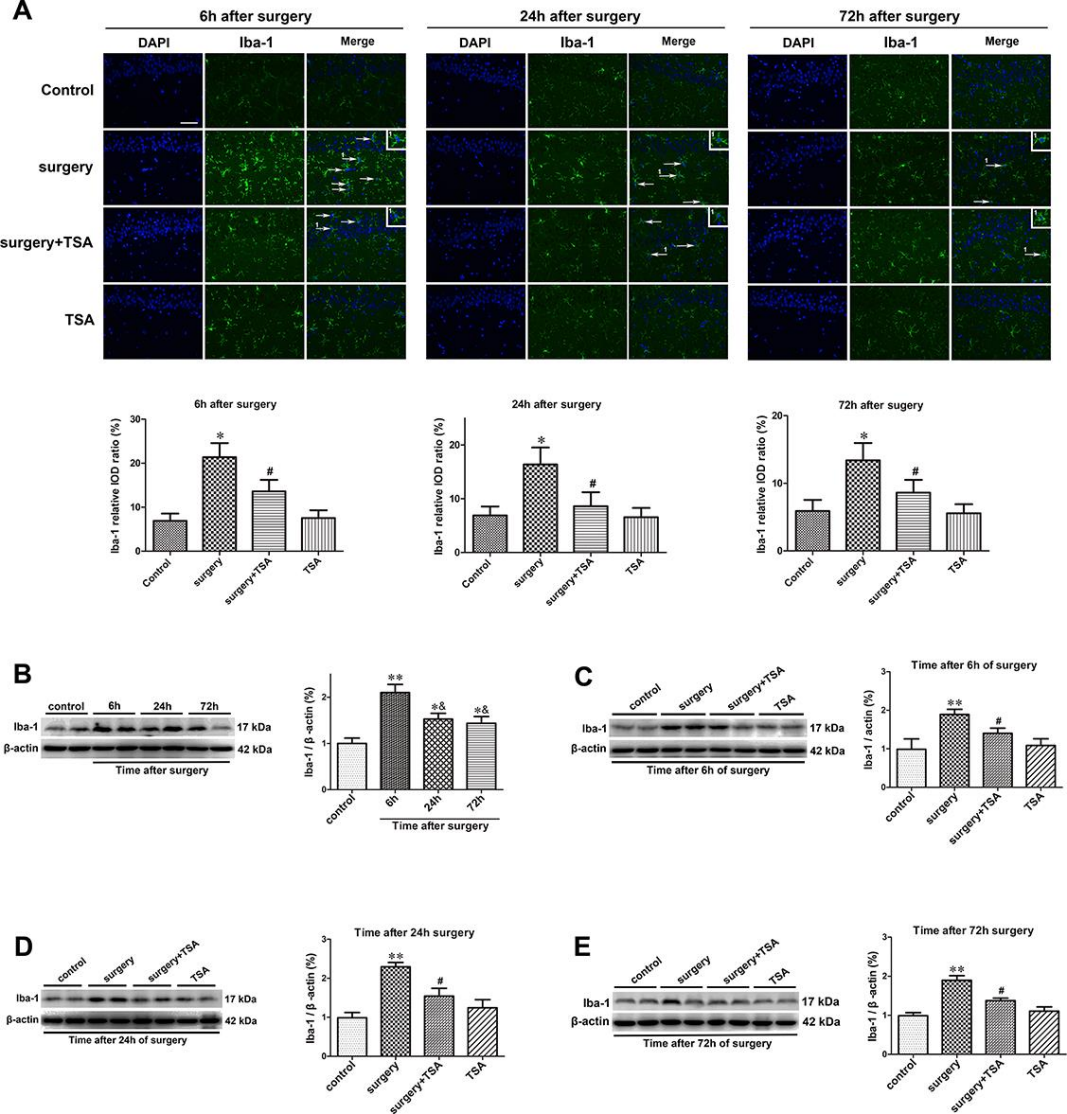
**TSA inhibits surgery-induced hippocampal microglia activation in aged rats.** (**A**) Immunofluorescence analysis and semi-quantification of higher expression of Iba-1 protein in the hippocampal CA1 area were observed at postoperative 6 h, 24 h and 72 h, and this staining was significantly inhibited by TSA pretreatment (Iba-1, green; cell nuclei, blue. Magnification 400 ×, Scale bar = 50 μm). Western blot analysis and semi-quantitative data showing protein expression (**B**–**E**), indicating that the expression levels of Iba-1, a microglia activation marker, increased significantly at 6 h, 24 h and 72 h after laparotomy, and it peaked at 6 h and decreased within 72 h post-surgery, which was significantly inhibited by TSA pretreatment, with β-actin used as a loading control. Data are given as means {plus minus} SEM, n = 5. *P < 0.05 and **P < 0.01 vs. the control group; #P < 0.05 vs. the surgery group; &P < 0.05 vs. 6 h after surgery.

Microglia mediate cytokine expression in the CNS, and limiting microglial activity is considered beneficial to reduce neuroinflammation [24]. The effects of TSA on surgery-induced microglial activation were determined. The results of the immunofluorescent examination showed that Iba-1 levels were significantly higher in the hippocampal CA1 region (arrowheads) of surgery-only challenged rats and were reduced in rats pretreated with TSA at 6, 24 and 72 h postsurgery (Figure 2A). The western blotting results confirmed that hippocampal Iba-1 protein expression peaked at 6 h and decreased within 72 h postsurgery compared with the controls (Figure 2B; *P* < 0.01 and *P* < 0.05). Again, Iba-1 protein expression was significantly higher in surgery-only-treated than in the control, surgery + TSA-treated and TSA-treated rats at 6, 24 and 72 h postsurgery (Figure 2C–2E; *P* < 0.01 and *P* < 0.05). Hippocampal Iba-1 protein levels were not altered following TSA-only treatment. These results indicated that surgery induced microglia activation and that this was prevented by TSA in aged rats.

### TSA inhibits surgery-induced neuroinflammation

**Figure 3:**
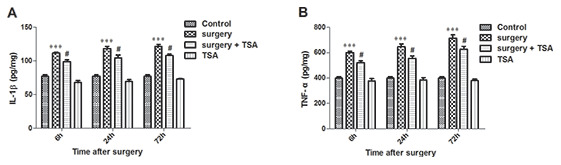
**Effects of laparotomy on the levels of hippocampal IL-1β and TNF-α in aged rats.** Compared with control rats, significant changes in the levels of IL-1β (**A**) and TNF-α (**B**) are observed at 6 h, 24 h and 72 h after surgery, which was significantly inhibited by TSA pretreatment. Data are given as means {plus minus} SEM, n = 5. ***P < 0.001 vs. the control group; #P < 0.05 vs. the surgery group.

TSA modulates cytokine synthesis and release [22]. We thus determined the effects of TSA on surgery-induced proinflammatory cytokine expression. Beginning at 6 h after surgery, the expression levels of IL-1β and TNF-α in the hippocampus were significantly increased until 72 h after surgery (Figure 3A, 3B, *P* < 0.001). In contrast, TSA treatment resulted in a significant reduction in IL-1β and TNF-α expression levels within 72 h, indicating that TSA reduced the inflammatory cytokines release in aged hippocampus induced by surgery.

### Proteomics profile of the selected regulated proteins in surgery-induced POCD rats

**Figure 4:**
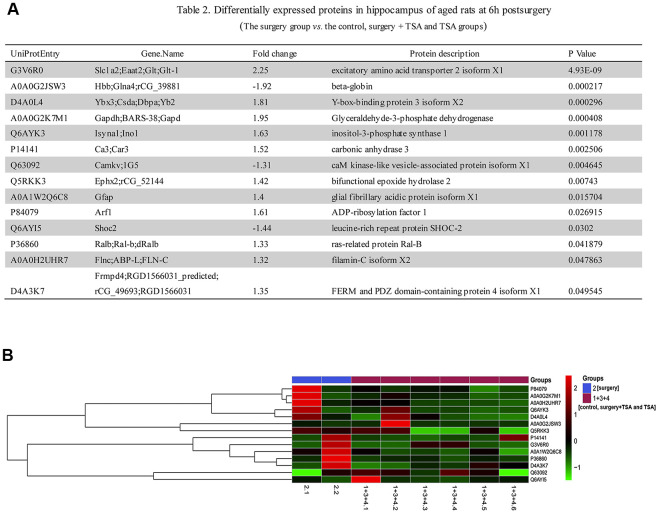
**Laparotomy alters hippocampal protein profile in rats.** (**A**) List of regulated proteins in hippocampus of aged rats at 6 h after surgery. (**B**) Hierarchical clustering of differentially regulated hippocampal proteins exhibiting their expressions in surgery group vs. control, surgery + TSA and TSA groups. Up-regulated protein expression values are displayed in red, the down-regulation values are in blue, and the intermediate values are in shades of red and blue.

To further understand the mechanism of surgery-induced POCD, we used iTRAQ to identify differentially expressed hippocampal proteins in the surgery group *vs.* control, surgery +TSA and TSA groups at 6 h after laparotomy (the peaking of neuroinflammation). Based on the iTRAQ-LC-MS/MS analysis results, fourteen quantified proteins with *P* < 0.05, a 1.2-fold cutoff was set to identify upregulated and downregulated proteins (Figure 4). Eleven categories differentially expressed proteins were upregulated, and three categories were downregulated after laparotomy.

### TSA inhibits surgery-induced neurofilaments (NFs) upregulation

**Figure 5:**
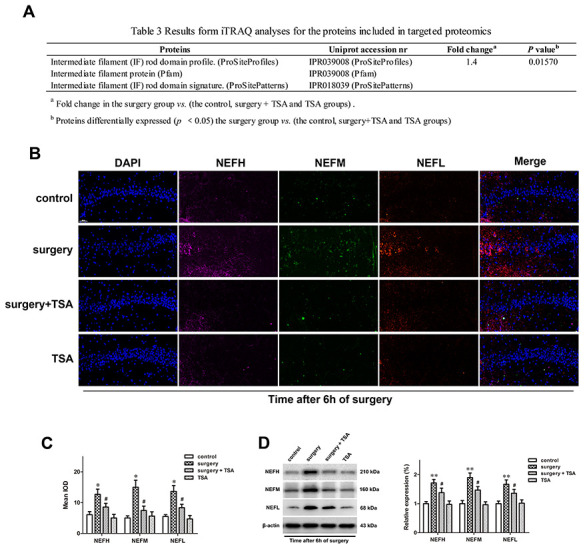
**TSA pretreatment reduces surgery-induced upregulation of NFs in the hippocampus of aged rats.** (**A**) iTRAQ analyses for the NFs proteins at 6 h post-surgery in proteomics. (**B**) Immunofluorescence analysis and (**C**) semi-quantification of the NFs reveals it increased in the hippocampal CA1 region at 6 h after surgery, which was significantly inhibited by TSA pretreatment (cell nuclei, blue; NEFH, purple; NEFM, green; NEFL, orange. Magnification 400×, Scale bar = 50 μm). (**D**) Representative western blotting images and statistical analysis of NEFH, NEFM and NEFL are shown. Data are given as means {plus minus} SEM, n = 4. *P < 0.05 and **P < 0.01 vs. the control group; #P < 0.05 vs. the surgery group.

Compared with the control, surgery + TSA and TSA groups, the results from the iTRAQ analyses of proteins in the surgery group, including the intermediate filament (IF) rod domain profile (ProSiteProfiles), IF protein (Pfam), and IF rod domain signature showed fold changes were greater than 1.4, and these proteins exhibited significant increases in IPR039008 (ProSiteProfiles), IPR039008 (Pfam) and PR018039 (ProSitePatterns) peptides (*P* = 0.01570) at 6 h after surgery (Figure 5A).

Based on the results of the MS analysis, the expression levels of three dysregulated proteins (NEFH, NEFM and NEFL) at 6 h after surgery were validated using immunofluorescence (Figure 5B, 5C) and western blotting (Figure 5D; *P* < 0.01 and *P* < 0.05). These three proteins were significantly upregulated in the surgery-treated group compared with the control group, and TSA pretreatment significantly prevented the surgery-induced increase in these proteins in the hippocampus. When given alone, TSA had no effect on the expression of NEFH, NEFM and NEFL at 6 h after surgery. Therefore, the altered expression levels of proteins were consistent with the results from the MS analysis.

### KEGG pathways analysis

**Figure 6:**
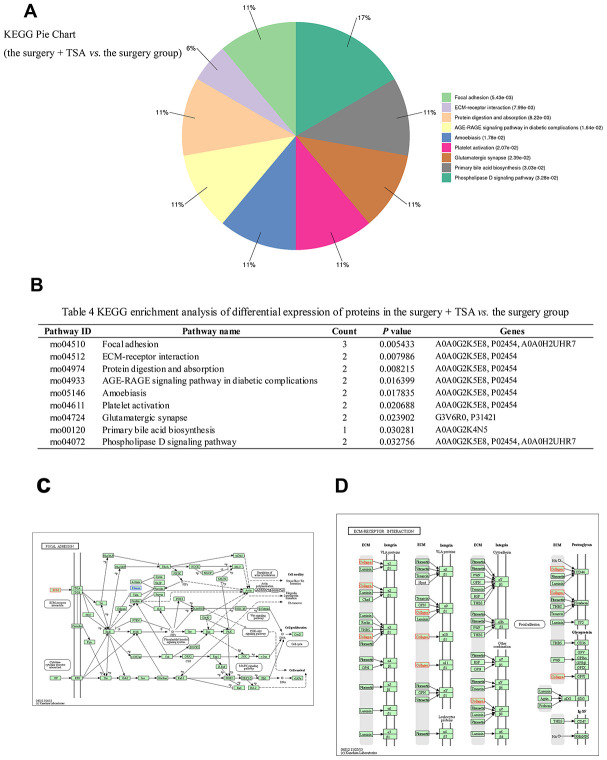
**KEGG pathway analysis in the surgery + TSA group vs. the surgery group at 6 h post-surgery.** (**A**) Top nine significantly enriched pathways identified by KEGG analysis. (**B**) Pathway enrichment analysis of differential expression of proteins. (**C**, **D**) Results show proteins involved in the focal eadhesion and ECM-receptor interaction pathways. Red colors represent the proteins that are upregulated, and blue colors represent the proteins that are downregulated.

An analysis of significantly differentially expressed proteins in the two groups was performed using the KEGG Pathway database (https://www.genome.jp/kegg/pathway.html). A total of 32 pathways were enriched in the surgery + TSA vs. the surgery group, and among them, nine pathways showed statistically significant enrichment (Figure 6A, 6B, *P* < 0.05), including focal adhesion (rno04510), ECM-receptor interaction (rno04512), protein digestion and absorption (rno04974), AGE-RAGE signaling pathway in diabetic complications (rno04933), amoebiasis (rno05146), platelet activation (rno04611), glutamatergic synapse (rno04724), primary bile acid biosynthesis (rno00120), and phospholipase D signaling pathway (rno04072). Of these, the “focal adhesion” (*P* = 0.005433) and “ECM-receptor interaction” (*P* = 0.007986) pathways were the top two significantly enriched pathways and were chosen for analysis. In Figure 6C, 6D, red represents upregulated proteins, and blue represents downregulated proteins.

## DISCUSSION

The laparotomy model was used in our study, which is a classic animal model widely used by researchers for POCD studies [10, 11]. Although accumulating evidence indicating that microglia activation and neuroinflammation played pivotal roles in the development of POCD [25, 26], little is known about its precise progression contributing to this postsurgical cognitive impairment. We have demonstrated that TSA pretreatment alleviates surgery-induced NFs damage in the hippocampus of aged rats by decreasing microglial activation and inflammatory factor (IL-1β and TNF-α) release, as well as changing the cargo of the proteins associated with the extracellular matrix (ECM). These results support the notion that microglial activation and its derived neuroinflammation play a key role in surgery-induced cognitive dysfunction. The results also provide some insight into the NFs damage triggered by neuroinflammation observed in the aged brain, which was suppressed by TSA through the “focal adhesion” and “ECM-receptor interaction” pathways. Our data explored the role of infiltrating immune cells in the progress of POCD. In neurodegenerative or neuroinflammatory diseases, the CNS exhibits increased activation of microglia and higher levels of proinflammatory cytokines such as TNF-α and IL-1β [27, 28], which is consistent with our results. These cytokines promote the release of secondary inflammatory mediators including prostaglandins and nitric oxide [29], which are essential for the induction and maintenance of the behavioral symptoms of this condition [30]. According to our results, the aged rats exhibited deficits in hippocampus-dependent learning and memory after surgery. TSA, which can cross the BBB [31], can alleviate expression of Iba-1, a marker of microglia activation, and reduce the levels of the proinflammatory cytokines IL-1β and TNF-α in aged rat brains, improving surgery-induced cognitive dysfunction.

Pathophysiology of POCD is very complicated, while iTRAQ analysis was performed on the hippocampus of aged rats at 6 h (the peak of neuroinflammation) after laparotomy to further explore the differentially expressed proteins in the POCD model. The identification, classification, and analysis of differentially expressed proteins should shed light on the molecular basis of surgery-induced cognitive dysfunction. There are 35 significant differentially expressed proteins and can been classified into 14 categories in this study. Several structural proteins including NFs are upregulated at 6h after laparotomy in hippocampus of aged rats. NFs, a type of IF, are polymers made from NFEL, NFEM, NFEH and α-internexin or peripherin; each subunit has various structural domains and functions [32]. Basal neurotransmission and induction of hippocampal long-term potentiation are abnormal in NFEM knockout mice, and NFEH knockout mice showed a markedly decrease in conduction velocity in large myelinated axons [33]. Moreover, NFEL was significantly increased in the plasma of Alzheimer's disease patients (149% vs. control) [16]. Our results show that surgery upregulated the expression of NEFH, NEFM and NEFL in aged rats (due to the degradation of NFs), thus reducing the NFs content, which was consistent with a published study [32]. Therefore, the calibers of large axons can exhibit general axonal dysfunction and decreased conduction velocities, leading to hippocampal dysfunction, which is characterized by spatial learning and memory declines. In this study, excessive activation of microglia were observed in the hippocampus after laparotomy, which might in turn trigger the release of IL-1β and contribute to NFs degradation [34]. The expression level of NFs in the surgery group was much higher than those in the TSA pretreatment and control groups, indicating that TSA could attenuate the NFs degradation in the hippocampus, which might play an important role in improving the surgically-induced cognitive dysfunction in aged rats.

Nine different KEGG pathways were altered in the TSA + surgery group compared with the surgery group. Moreover, the reactome pathway analysis showed a predominance of proteins involved in the “focal adhesion” and “ECM-receptor interaction” pathways, which are related to NFs degradation and the aggregation phase in the hippocampus after laparotomy, as evidenced by marked collagen upregulation and filamin downregulation (Figure 6A, 6B). The present results showed that TSA clearly increased collagen protein in the hippocampus. Collagen is the main component of the ECM and provides the structural support that is required for normal ECM assembly, and it suppresses NF overexpression in response to surgery (Figure 6C, 6D) via activation of the “focal adhesion” and “ECM-receptor interaction” signaling pathways. Thus, these two pathways may be involved in the improving of TSA in surgery-induced spatial memory impairment. We hypothesize that the accumulation of collagen might provide structural support to myelinated axons in the hippocampus and alleviate the degradation of NFs, protecting the normal conduction function of axons and increasing neuronal efficiency in the damaged circuit. However, this is only informed speculation as collagen has been previously reported to be one of the main materials that promotes repair of damaged nerves [35] and guides newborn NFs extension [36], thus enhancing nerve regeneration and functional recovery. In summary, the role of significantly differentially expressed signaling pathway proteins in the improvement of cognitive dysfunction is not necessarily proportional to these differences, and further verification of which signaling pathway is more effective in improving surgery-induced POCD is urgently needed.

## CONCLUSIONS

This study suggests that surgery-induced neuroinflammation is associated with neural damage in aged rats, including elevated levels of structural proteins such as NFs in the hippocampus, and contributes to the spatial memory impairment. A single administration of TSA before laparotomy effectively prevented spatial memory impairment of aged rats. The potential mechanism of TSA was suppressing microglial activation and inflammatory cytokine release, causing collagen overexpression thus protecting the normal structure and conduction function of NFs in the hippocampus. This function is mainly attributed to the activation of two important signaling pathways, the “focal adhesion” and “ECM-receptor interaction” signaling pathways.

## MATERIALS AND METHODS

### Animals

Aged male Sprague-Dawley rats (20 months old; weight, 550–650 g) were used for all experiments. Animals were purchased from the Dongchuang Laboratory Animal Center (Changsha, Hunan, China) and bred under standardized housing conditions with ad libitum access to food and water. All rats were allowed to adapt to the new environment for at least 7 days before experiments. The experimental protocol was approved by the Institutional Animal Care and Use Committee of the Sixth People’s Hospital Affiliated with Shanghai Jiao Tong University (SYXK [Shanghai, China] 2016-0020, 22 February 2017).

### POCD Model

Animals were exposed to 1.5% isoflurane for 5 min in a small chamber and then removed and endotracheally intubated [37]. The laparotomy was aseptically performed under mechanical ventilation using a previously described method (1%–2% isoflurane in 100% oxygen) that was developed to model POCD in aged rats [10]. Briefly, with the surgeon wearing sterile latex gloves, the abdominal region was shaved and sterilized. A 3-cm vertical incision was made approximately 0.5 cm below the lower right rib. The viscera and incised muscle were vigorously manipulated by inserting an index finger up to the second knuckle into the opening for 30 s. Next, approximately 10 cm of the intestine was exteriorized and vigorously rubbed with the thumb and index finger, also for 30 s, and then placed back into the cavity. Finally, the surgeon separately sutured the peritoneal lining, abdominal muscle and skin. The laparotomy duration was 20–25 min. The sham operation group was treated in an identical manner for the same amount of time, except that laparotomy was not performed.

### Experimental protocol

### Effect of TSA block on spatial learning and memory

The effects of TSA (Sigma, St, Louis, MO) on surgery-induced (laparotomy) cognitive decline were examined. Rats were randomly assigned to control, surgery, surgery + TSA and TSA groups (n = 12 each). Rats in the surgery + TSA and TSA groups were intraperitoneally administered 1 mg/kg TSA 30 min before surgery; this dosing protocol has been shown to effectively protect against kainic acid-induced memory deficits [31]. TSA was dissolved in a vehicle solution (1 mg/ml in 10% DMSO). Rats in the other two groups received an identical volume of vehicle solution. Following the pretreatment phase, the animals either underwent laparotomy or sham surgery under isoflurane anesthesia.

The Morris Water Maze (MWM) test was used to evaluate the learning and memory of aged rats. The MWM test was performed 2 days after surgery (allowing for abdominal incision healing) and conducted by investigators blinded to the group conditions as previously described [11]. Swimming was tracked by video (Sunny Instruments Co. Ltd., Beijing, China). The latency, swim speed, time to first platform crossing and time spent in the previous platform quadrant were analyzed.

### Effects of laparotomy on physiological parameters in aged rats

To determine whether isoflurane anesthesia and laparotomy caused physiological side effects such as hypoxia, hypercapnia or hypoglycemia, five rats in the various treatment groups were selected as cardiorespiratory control animals (total: n = 20). After the surgery, blood samples (0.5 ml) were immediately collected for arterial blood gas (OPTI Medical Systems, Roswell, GA) and blood glucose (Life Scan Inc., Milpitas, CA) analysis. The cardiorespiratory control rats were not used for any other part of the study.

### Neuroinflammation and iTRAQ-based proteomics in the hippocampus after surgery

To study the effects of peripheral surgical trauma on microglia activity in the brain, rats were randomly assigned to control, surgery, surgery + TSA and TSA groups and received laparotomy or sham operations. Markers for microglial activation in the hippocampus were determined at 6, 24 and 72 h after surgery using western blotting and immunofluorescence (n = 5 per time point).

**Figure 7:**
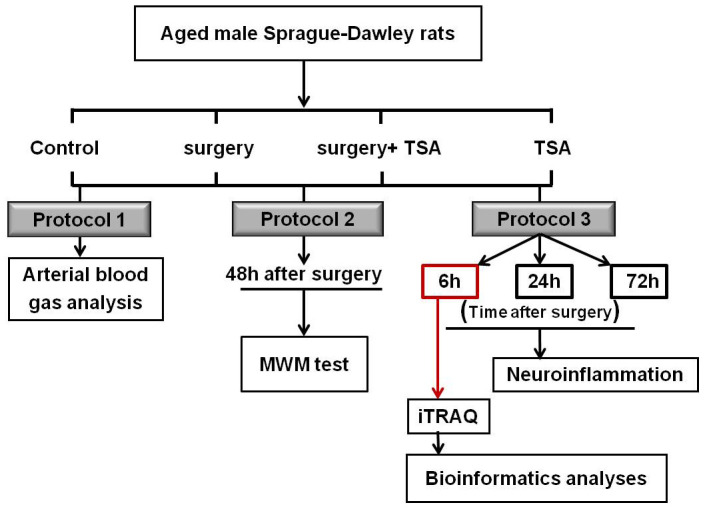
**Experimental schedule.** Aged rats were divided into four groups: control, surgery, surgery+ TSA and TSA. Rats in the surgery + TSA and TSA groups were intraperitoneally administered 1 mg/kg TSA 30 min before surgery or sham under isoflurane anesthesia. Protocol 1: After the surgery, blood samples (0.5 ml) were immediately collected for arterial blood gas and blood glucose analysis. Protocol 2: Spatial learning and memory were then tested using the Morris water maze (MWM) task on day 2 postsurgery. Protocol 3: Markers for microglial activation and some of inflammatory cytokine in the hippocampus were determined at 6, 24 and 72 h after surgery, respectively. At the peak of neuroflammation we have observed, at 6 h postsurgery, the protein profile alterations in the hippocampus were assessed using iTRAQ. Then, bioinformatics analysis was performed. Surgery: abdominal exploratory surgery; TSA: Trichostatin A.

In the present study, we found that expression of Iba-1 (a microglial cell activation marker) was significantly increased at 6 h after laparotomy compared with other observational time points, indicating that this marker peaked at 6 h after surgery; therefore, the effects of surgery on the protein profile alterations in the hippocampus were assessed at 6 h after surgery using iTRAQ (n = 4 each). The experimental schedule is shown in Figure 7.

### Enzyme-linked immunosorbent assays (ELISAs)

Expression of proinflammatory cytokines in the hippocampus was determined with an ELISA (IBL, Takasaki, Japan). The hippocampus was separated, homogenized in extraction buffer and centrifuged, and the total protein concentration of the supernatant was determined using a bicinchoninic acid (BCA) protein assay kit. Then, 100 μl of the supernatant was collected and analyzed by an ELISA according to the manufacturer’s instructions. The results were assayed at 450 nm, and data are expressed as pg/mg of tissues.

### Western blot

Western blots were performed as previously described [2]. The proteins (30-50 μg) were transferred into polyvinylidene fluoride microporous membranes. Then, the membranes were blocked with 5% non-fat milk in TBST for 2 h at room temperature and incubated overnight on lab shaker at 4° C with the following primary antibodies: anti-Iba-1 (1:1000; Abcam, San Diego, CA); anti-neurofilament (NF) light chain (anti-NEFL) (1:1000; Abcam); anti-NF medium chain (anti-NEFM) (1:1000; Abcam); and anti-NF heavy chain (anti-NEFH) (1:1000; Abcam). Fluorescently labeled secondary antibodies (1:10,000; LI-COR Biosciences, Lincoln, NE) were also used.

### Immunofluorescence

Immunofluorescence staining was performed as previously described [2, 24]. The brains were harvested after surgery and were dissected and incubated with 4% paraformaldehyde for 24 h, then dehydrated in 30% sucrose solution for 48 h until sinking to the bottom. Twenty micrometer-thick coronal hippocampal cryosections were cut via cryostat (Leica, Wetzlar, Germany). Then sections were incubated with the primary antibody, including anti-Iba-1 (1:1,000; Abcam), anti-NEFL (1:500; Abcam), anti-NEFM (1:500; Abcam), and anti-NEFH (1:100; Abcam), the signal was detected with a fluorescein isothiocyanate-labeled secondary antibody (1:200; Abcam). The nuclei were counterstained with 4, 6-diamidino-2-phenylindole (DAPI) (1:5,000; Roche, Mannheim, Germany). Images were acquired on a Leica DM3000 fluorescence microscope (Leica, Wetzlar, Germany). Semi-quantitative analysis was performed at 400× magnification per visual field (0.145 mm^2^) for expressions of Iba-1, NEFL, NEFM and NEFH, using imaging software (ImagePro Plus 6.0; Media Cybernetics, Bethesda, MD, USA). The mean IOD values were analyzed and averaged.

### iTRAQ labeling and NanoLC-MS/MS analysis

Hippocampus tissues were ultrasonically disrupted in lysis buffer (Roche) on ice. Supernatants were collected after centrifugation (10,000 g, 30 min, 4° C), and protein concentrations were determined using an enhanced BCA Protein Assay Kit (P0010; Beyotime Biotechnology Ltd., Beijing, China) following the manufacturer’s instructions. The protein samples (200 μg) were mixed with dl-dithiothreitol, alkylated with iodoacetamide and then digested in trypsin (protein-trypsin ratio = 50:1, 12 h). Then, the peptides were labelled with an iTRAQ reagent-8-plex multiplex kit according to the manufacturer’s instructions. Samples were labeled with the iTRAQ tags as follows: the control group (tags 113 and 117), the surgery group (tags 115 and 119), the surgery + TSA group (tags 116 and 121) and the TSA group (tags 114 and118). All labelled samples were mixed and dried by vacuum centrifugation (EYELA, Tokyo, Japan).

The peptides were re-dissolved in 30 μl of solvent A (A: 0.1% formic acid in water) and analyzed by on-line nanospray LC-MS/MS on an Orbitrap Fusion™ instrument coupled to an EASY-nLC 1200 system (Thermo Fisher Scientific, MA, USA). The peptide sample (4 μl) was loaded (trap column (Thermo Fisher Scientific Acclaim PepMap C18, 100 μm x 2 cm), analytical column (Acclaim PepMap C18, 75 μm x 15 cm)) and separated with a linear gradient, ranging from 3% B (B: 0.1% formic acid in ACN) to 32% B in 120 min. The column flow rate was maintained at 300 nl/min with a column temperature of 40° C. An electrospray voltage of 2 kV vs. the inlet of the mass spectrometer was used.

The mass spectrometer was run in the data-dependent acquisition mode and automatically switched between the MS and MS/MS mode. The parameters were as follows: (1) MS: scan range (m/z) = 350–1550; resolution = 60,000; AGC target = 4e5; maximum injection time = 50 ms; included charge states = 2–6; dynamic exclusion = 45 s; (2) HCD-MS/MS: resolution = 30,000; isolation window = 1.2; AGC target = 7e4; maximum injection time =100 ms; collision energy = 38.

### MS data analysis

Tandem mass spectra were processed by PEAKS Studio version 8.5 (Bioinformatics Solutions Inc., Waterloo, Canada). PEAKS DB was set to search the UniProt-Rat database (30226 entries, ver 201708) using trypsin as the digestion enzyme. The PEAKS DB search was performed with a fragment ion mass tolerance of 0.05 Da and a parent ion tolerance of 7 ppm. Carbamidomethylation (C) and iTRAQ 8plex (K, N-term) were specified as the fixed modifications. Oxidation (M), Deamidation (NQ), and Acetylation (Protein N-term), were specified as the variable modifications. Peptides were filtered with a 1% FDR, and a unique peptide was specified. PEAKSQ was used to calculate peptide and protein abundance. Normalization was performed when averaging the abundance of all peptides. Medians were used for averaging. Differentially expressed proteins were filtered if their fold change was greater than 1.2 and they contained at least two unique peptides with a significance value greater than 13 (*P* < 0.05).

### Bioinformatics analysis

Blast2GO version 4 was used for functional annotation. The whole protein sequence database was analyzed by BlastP, and the results were mapped and annotated with the Gene Ontology database. Functional statistics of differentially expressed proteins were calculated by Fisher’s exact test in Blast2GO. Hierarchical cluster analysis (HCA) is an algorithmic approach to find discrete groups with varying degrees of (dis) similarity in a data set represented by a (dis) similarity matrix. This analysis is processed with pheatmap package (https://CRAN.R-project.org/package=pheatmap) Pathway analysis was performed using the Kyoto Encyclopedia of Gene and Genomes (KEGG) and was processed by KOBAS (http://kobas.cbi.pku.edu.cn/). Functional protein association networks were generated using STRING.

### Statistical analysis

Statistics were calculated using SPSS 16.0 for Windows (SPSS, Inc., Chicago, IL, USA). Data on escape latency in the MWM tests were analyzed with two-way repeated-measures ANOVA followed by a post-hoc Bonferroni test. All other quantitative data were analyzed using one-way or two-way analysis of variance (ANOVA) followed by the Bonferroni post hoc test. Statistical significance was set at *P* < 0.05. All data are shown as means ± SEM (standard error of the mean).

### Availability of data and materials

All data generated or analyzed during this study are included in this published article.

### Ethics approval and consent to participate

Our research was performed with the approval of the Sixth People’s Hospital Affiliated with Shanghai Jiao Tong University Biomedical Ethics Committee Experimental Animal Ethics Branch (SYXK [Shanghai, China] 2016-0020, 22 February 2017).
